# Clinical movement disorders – a new era

**DOI:** 10.1186/2054-7072-1-1

**Published:** 2014-10-29

**Authors:** Steven J Frucht

**Affiliations:** Department of Neurology, Icahn School of Medicine at Mount Sinai, New York, New York USA

We are proud to announce the launch of a new open access journal, entitled *Journal of Clinical Movement Disorders* (*JCMD*). The aim of the journal is to offer an open access forum for the publication of scientifically rigorous articles focusing on the natural history, etiology, evaluation and treatment of patients with a wide range of movement disorders [1]. We are interested in topics important to the practicing clinician, but especially in articles that explore the ways in which translational approaches are changing the evaluation and treatment of patients with movement disorders in the 21^st^ century.

We are particularly pleased to launch *JCMD* as a fully open access journal. At present, only one journal devoted to movement disorders (*Tremor and Other Hyperkinetic Disorders*) offers an open access format. We believe that the open access format offers numerous advantages to traditional publication strategies. Articles will be published as soon as they are accepted, markedly decreasing the time for evaluation and distribution. All articles are available online at no cost throughout the world, enabling the widest possible reach for the journal irrespective of a reader’s location or economic resources [2]. Authors will continue to hold copyright for their work, and articles will be archived in PubMed Central. The online format allows articles to be published without constraints on length of text. Further, the format is a perfect vehicle for publication of videos demonstrating salient features in articles, a critical feature of clinical movement disorders.

*JCMD* will publish articles covering the full spectrum of movement disorders including hypokinetic disorders (Parkinson’s disease, atypical parkinsonian disorders such as multiple system atrophy, progressive supranuclear palsy, corticobasal syndrome, dementia with Lewy body disease, frontotemporal dementia, etc.), and usual and unusual hyperkinetic disorders (myoclonus, chorea, tics, tremor, dystonia, stereotypies, paroxysmal movement disorders, etc.). We are interested in both descriptive articles that illustrate phenotype, as well as hypothesis driven articles that explore the etiology, genetics, pharmacology, neuroimaging and treatment options for these patients. Submissions on both adult and pediatric disorders are welcome, and we are particularly interested in novel treatments such as deep brain stimulation. Special features that we plan to integrate into the journal include a clinical phenomenology section, a section devoted to current controversies, and reviews that summarize important findings in the field.

All manuscripts submitted to *JCMD* will be independently reviewed by two experts in the field, by a member of the Editorial Board and by the Editor-in-Chief. We are interested in publishing articles of the highest quality and novelty, but will also consider research that may be of more general interest to readers due to novel clinical or examination features, clinical relevance or unusual phenotype, even if the findings are confirmatory. We believe that clinical movement disorders is unique as a subspecialty area in neurology, in that many are drawn to the field by the complexity and subtlety of the clinical examination, and the way in which 21^st^ century technologies can help illuminate these findings.

The Editorial Board of *JCMD* includes 43 members from 11 different countries [3]. We believe that the broad expertise and international reputations of the Editorial Board attests to their confidence that *JCMD* will succeed. We hope that you enjoy *JCMD*, and that you will consider it when deciding where to publish your work.

Welcome!
